# Drug-micronutrient interactions: food for thought and thought for action

**DOI:** 10.1186/s13167-016-0059-1

**Published:** 2016-05-12

**Authors:** Vasiliki Karadima, Christina Kraniotou, George Bellos, George Th. Tsangaris

**Affiliations:** 1 Koropi Health Centre, 19400 Attica, Greece; 2 Proteomics Research Unit, Biomedical Research Foundation of the Academy of Athens, Athens, Greece

**Keywords:** Predictive preventive personalized medicine, Micronutrients, Micronutrient deficiency, Epidemiology, Drug-nutrient interactions, Multi-professional network, Advanced health care, Well-being

## Abstract

Micronutrients are indispensable for a variety of vital functions. Micronutrient deficiencies are a global problem concerning two billion people. In most cases, deficiencies are treatable with supplementation of the elements in lack. Drug-nutrient interactions can also lead to micronutrient reduce or depletion by various pathways. Supplementation of the elements and long-term fortification programs for populations at risk can prevent and restore the related deficiencies. Within the context of Predictive, Preventive, and Personalized Medicine, a multi-professional network should be developed in order to identify, manage, and prevent drug-micronutrient interactions that can potentially result to micronutrient deficiencies.

## What are micronutrients?

Micronutrients [1] is a term generally used to define all essential vitamins and minerals mainly taken from food sources and which are necessary for vital functions [2, 3]. Micronutrients consist only of 0.01 % of body mass [1]. Surprisingly, even if the amounts required are very low, a lack of micronutrient can lead to severe, non-ignorable health disorders, even threatening for life [2]. Fortunately, most of these dysfunctions can disappear after the administration of the elements in lack [3].

## What about epidemiology?

Micronutrient deficiencies (MNDs) are a very common condition. It is estimated that about two billion people in the world suffer from MNDs [2]. Notably, this is not a problem of developing countries exclusively [4]. Many people in west societies are diagnosed with MNDs [4]. However, in these countries, MNDs often remain undiagnosed and are supposed to affect 1 in 3 persons [3]. Malnutrition is the major cause of MNDs [3], and it may include low intake or malabsorption of micronutrients owing to infection, inflammation, or a systematic disease [2]. Pregnant women, children less than 5 years of age, and elderly people are more likely to suffer from any type of MNDs [2]. The most frequent deficiency is that of iron [1], followed by vitamin A, folate, iodine, and zinc deficiency [3]. In minors, vitamin and folate deficiencies dominate across all age groups [4]. Appearance of multiple MNDS is more common than that of sole [2]. Table 1 describes selected micronutrient deficiencies and their clinical manifestations and diagnosis.

**Table 1:** Selected micronutrients: role, signs, and symptoms of deficiencies and accurate diagnosis

Micronutrient	Functions	Symptoms and signs of deficiency	Diagnosis
Iron	Constituent of hemoglobin, carries out oxygen transport, indispensable for cognitive functions	Anemia, endocrine and immune disorders, ↑ danger for maternal death Newborns: ↓ birthweight, prematurity, perinatal complications, physical and mental retardation	At least 2 of 3: ↓ Hemoglobin ↓ Ferritin ↓ Transferrin saturation
Vitamin A	Participates in vision, immunization, reproduction, growth	Sensitivity, infections, xerophthalmia and other vision problems, blindness in children	↓ Serum retinol, ophthalmologic examination
Iodine	Constituent of thyroid hormone, CNS growth in fetus and infant	Fetus: neurological and mental retardation (permanent), cretinism Adult: goiter, ↓ mental function, hypo/hyperthyroidism	↓ Urine iodine
Folate	Constituent of vitamin B, participates in DNA synthesis, stability, and repair, disinclines mutations	Megalosblastic–macrocytic anemia Fetus: neural tube defects	↓ Concentration in serum, plasma, and erythrocytes
Zinc	Activates enzymes involved in immunization, necessary for fetus and children growth	↑ Morbidity and mortality of diarrhea, respiratory infection, and malaria	No reliable biomarker due to ↓ bio-ability

For example, iron is an essential element for oxygen transportation, the red blood cells, and several enzymes’ production and important immune functions. Its deficiency that affects millions lies hidden of the overall death rates, maternal hemorrhage, reduced mental, and physical performance. Reduced levels of blood hemoglobin, serum ferritin, and low transferrin saturation confirm the diagnosis of iron deficiency

## But how micronutrient status can be determined?

Modern methods are currently used to count the amount of a micronutrient in the body. The most accurate method is metabolites biomarkers [1], which count micronutrient levels using blood or urine samples [2]. The functional intracellular analysis—within lymphocytes in blood samples or buccal mucosa cells—is a novel (part of -omics science) reliable process of micronutrient testing [5–7].

## How MNDs can be faced?

MNDs can be prevented massively by the fortification of elements in lack in a country or region. Preventive programs are often applied to population at risk. The most common example is the enrichment of table salt carried forward by many governments. On the other hand, population groups at risk may receive supplementation according to guidelines. For instance, iron and folic acid are prescribed to pregnant women [2].

In modern societies, the increase of life-span leads to multi-morbidity and inevitably to polypharmacy. Polypharmacy added to inappropriate drug prescribing increases the risk of drug-drug and drug-nutrient interactions (DNIs) [8–10]. DNIs are not rare, with the potential for over 300 remedies capable of interacting with nutrient or food components [11]. MNDs can be the adverse effect of these interactions, especially in elder and chronically ill people with impaired nutritional status.

## Drug-nutrient interactions

The term DNIs refers to physicochemical, physiological, or pathophysiological relationships between a drug and a nutrient [12] or, in a broad sense, between a drug and multiple nutrients, food or components, or nutritional status [13, 14]. DNIs can be classified in four types: *type I*, ex vivo bio-inactivation; *type II*, decreased/increased absorption; *type III*, decreased/increased effect; and *type IV*, decreased/increased clearance [8, 9, 12].

**Fig. 1:**
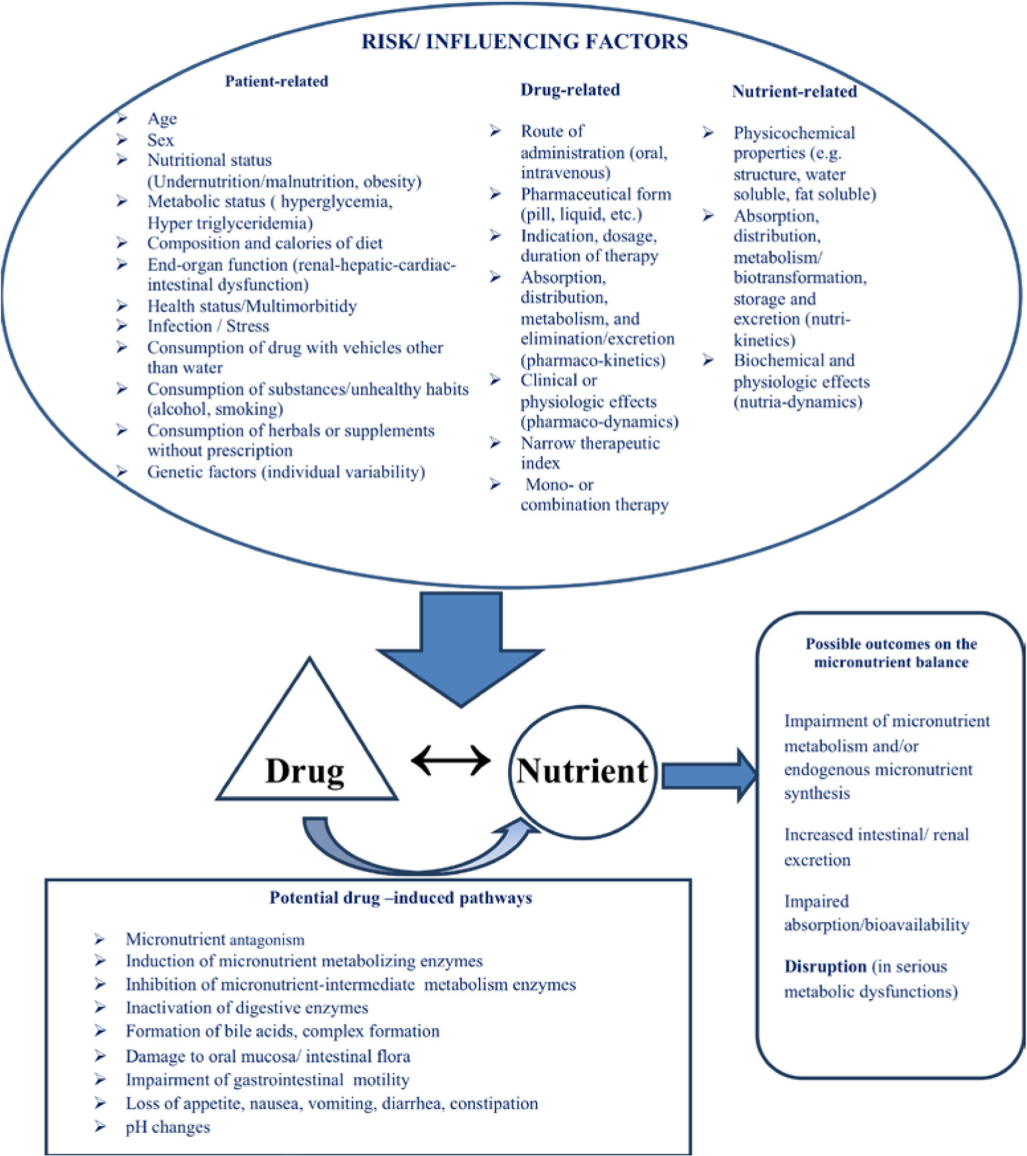
Drug-nutrient interactions: influencing factors, possible pathways, and potential effects on the micronutrient balance. Risk for drug-nutrient interactions can be affected by many precipitating/influencing factors. These factors are related to individual characteristics (e.g., age, gender, medical history, genetic profile), remedies (pharmacokinetics, pharmacodynamics), and micronutrients properties (nutrikinetics, nutridynamics). Frequently, DNIs are bidirectional in their outcomes. Drugs can potentially influence the metabolism of micronutrients in multiple ways, from their intestinal absorption to their cell bioavailability. Intermediate pathways include a wide range of alterations in physiological processes such as increase in gastrointestinal motility (e.g., induced from metoclopramide, erythromycin, and cisapride) and thus limited absorption and increased nutrient loss. As a result, the micronutrient balance is affected and elimination may occur in severe and prolonged DNI, when a drug impairs/inhibits micronutrient absorption or metabolic functions

A clinically significant drug-nutrient interaction is one related to an impaired physiologic process (quantifiable alteration of the kinetic and/or dynamic profile of a drug or a nutrient), which may result to malnutrition, therapy failure, adverse events, or even a life-threatening situation [8, 13]. Factors that may enhance the type and intensity of DNIs include patient-related variables as age, sex, comorbidities, nutritional status, and also drug- and nutrient-associated factors as route of administration, nutrient status, and pharmacological/toxicological profile of the drug [8, 9, 13] (Fig. 1). Some of the effects that commonly used drugs can have on micronutrient homeostasis are described in the Table 2.

**Table 2:** Drug-micronutrient interactions: widely used categories and possible drug-induced pathways that lead to nutrient depletions

Widely used drugs (category)	Proposed mechanisms	(Micro) nutrient depleted
Acid-suppressing and antacids	↓ Absorption	H2 antagonists Calcium, iron, zinc, folic acid, vitamin D, and vitamin B_12_ Proton-pump inhibitors (PPIs) Vitamin B_12_ and magnesium
Antibiotics	↓ Absorption Complex formation Chelation Enzyme induction Mucosal block/damage ↓ Endogenous production	Folic acid, iron, vitamin A, vitamin D, B_1_ (thiamin), B_2_ (riboflavin), B_3_, B_6_, B_12_, calcium, magnesium, potassium, and vitamin K
Anti-hypertensives	↓ Cell availability ↑ Renal elimination	Angiotensin-converting enzyme inhibitors: zinc Calcium channel blockers: potassium Chlorthalidone, hydrochlorothiazide, zinc, potassium, B vitamins Loop diuretics: calcium, magnesium, potassium, zinc, vitamins B_1_ and B_6_ Hydralazine: vitamin B_6_ and coenzyme Q_10_ Beta-blockers: coenzyme Q_10_ Potassium-sparing diuretics: folic acid
Antiepileptic drugs (anti-convulsants)	↓ Absorption ↑ Metabolism Enzyme induction Chelation	Barbiturates: calcium, folic acid, vitamins D and K Phenytoin: calcium, folic acid, vitamins B_1_, B_2_, and D Carbamazepine: folic acid and vitamin D Valproic acid: l-carnitine
Psychotherapeutic drugs	Enzyme induction ↑ Metabolism ↓ Endogenous production	Selective serotonin reuptake inhibitors (SSRIs): folic acid Benzodiazepines: melatonin, calcium Tricyclic antidepressants, phenothiazines: coenzyme Q_10_ and vitamin B_2_ Haloperidol: coenzyme Q_10_
Cholesterol-lowering drugs: statins	↓ Cell availability ↓ Endogenous production	Coenzyme Q_10_, vitamin D
Digoxin	↑ Renal elimination	Magnesium, potassium, calcium, phosphorus, vitamin B_1_
Oral hypoglycemics	↓ Absorption	Metformin: vitamin B_12_
Oral contraceptives	↓ Absorption Enzyme induction	Vitamin B_6_, folic acid, magnesium
Hormone replacement therapy (estrogens)	↓ Absorption ↑ Metabolism ↑ Excretion	Vitamin B_6_, folic acid, magnesium
Anti-inflammatory/analgesics	↓ Absorption ↓ Cell availability	Non-steroidal anti-inflammatory drugs: iron and folic acid Salicylate: iron, folic acid, potassium, sodium, and vitamin C

For example, *diuretics* (anti-hypertensives) will possibly lead to a loss of micronutrients, especially of the water-soluble vitamins (vitamin B) and minerals (K, Mg, Ca), due to renal hyper-excretion. Some *antibiotics* can reduce the vitamin K synthesis by intestinal bacteria. *Metformin*, after long-term therapy, is associated with reduced vitamin B_12_ levels by decreasing the uptake of B_12_ via calcium-dependent ileal cell membrane receptors and thus affected absorption

## How DNIs can be addressed?

Physicians must coordinate with nutritionists, nurses, and pharmacists in order to minimize DNIs and adverse outcomes. Α comprehensive strategy can be planned, based on their knowledge, experience, and skills. Awareness of drug interactions with common dietary agents, defined drug administration schedules, periodic review of current drug therapy and dietary habits, proper education of health-care providers, and computerized drug interaction screening and warning software combined with patient counseling are crucial steps of this innovative approach [9, 10, 15].

## Conclusion

MNDs can be the result of malnutrition or the adverse outcome of common DNIs. In order to handle effectively MNDs and DNIs, modern health-care services should be governed by the principles of PPPM. PPPM uses advanced science technologies (genomic, proteomic, and metabolomics biomarkers or bio-predictors) that allow to determine individual *predisposition* to a particular illness and prevent clinically established dys-homeostasis, by using *personalized*, preventive, and therapeutic strategies [16, 17]. According to the National Institute of Health (NIH) and other health authorities (European Commission, US Food and Agriculture Association (FDA), Centers for Disease Control and Prevention (CDC)), PPPM constitutes a fundamental crucial axis of development in the twenty-first century [16]. The *predictive* branch of PPPM includes the identification and evaluation of new biomarkers/bio-predictors in subclinical stages of the pathological process before the onset of clinical manifestations [16, 17]. Pharmacogenomics and nutrigenomics are new research fields that study gene-drugs and gene-nutrient interactions, aiming for the development of safe and effective drug-based therapies and the selection of health-promoting nutrients for *individuals* [18]. Accumulating data about the molecular mechanisms of DNIs will help us to generate a novel drug-diet interactome map and thus to identify, predict, and prevent possible unwanted interactions between natural compounds and drugs [19]. *Preventive* measures that include daily intake of micronutrients, accorded to health authorities’ recommendations [20], supplementation of the elements in lack, and long-term fortification programs for populations at risk can *prevent* and restore the related deficiencies. In addition, high-risk patients (elders, obese, critically ill, with chronic diseases, with known genetic variants in drug transporters, receptors, or enzymes) and individuals under high-risk medication (antimicrobials, antiepileptics, warfarin, drugs with narrow therapeutic index) should be targeted for DNI *monitoring* [15, 16]. The physician’s decision to screen for MNDs should be based on the patient’s history, comorbidities, dietary habits, and lifestyle. The aim of *personalized* medicine is the tailoring of health-care services to the needs of the individual patient and/or to the person-at-risk by the evaluation of integrated health data (family history, medical data, -omics profiles) [16, 17, 21]. Such holistic strategies can be applied and support appropriate drug prescribing and nutritional advices, in order to minimize DNIs and MNDs, reduce health-care utilization and costs, and enhance well-being.

## Abbreviations

MNDs: micronutrient deficiencies
DNIs: drug-nutrient interactions
PPPM: Predictive, Preventive, and Personalized Medicine
